# Survivorship Care Plan Information Needs: Perspectives of Safety-Net Breast Cancer Patients

**DOI:** 10.1371/journal.pone.0168383

**Published:** 2016-12-16

**Authors:** Nancy J. Burke, Tessa M. Napoles, Priscilla J. Banks, Fern S. Orenstein, Judith A. Luce, Galen Joseph

**Affiliations:** 1 University of California, Merced, School of Social Sciences, Humanities, & Arts, Merced, California, United States of America; 2 University of California, San Francisco, Department of Anthropology, History, and Social Medicine, San Francisco, California, United States of America; 3 Helen Diller Family Comprehensive Cancer Center, University of California, San Francisco, San Francisco, California, United States of America; 4 Breast Care Disparities Program, San Francisco General Hospital, San Francisco, California, United States of America; 5 School of Medicine, University of California, San Francisco, San Francisco, California, United States of America; Northwestern University Feinberg School of Medicine, UNITED STATES

## Abstract

**Purpose:**

Despite the Institute of Medicine’s (IOM) 2005 recommendation, few care organizations have instituted standard survivorship care plans (SCPs). Low health literacy and low English proficiency are important factors to consider in SCP development. Our study aimed to identify information needs and survivorship care plan preferences of low literacy, multi-lingual patients to support the transition from oncology to primary care and ongoing learning in survivorship.

**Methods:**

We conducted focus groups in five languages with African American, Latina, Russian, Filipina, White, and Chinese medically underserved breast cancer patients. Topics explored included the transition to primary care, access to information, knowledge of treatment history, and perspectives on SCPs.

**Results:**

Analysis of focus group data identified three themes: 1) the need for information and education on the transition between “active treatment” and “survivorship”; 2) information needed (and often not obtained) from providers; and 3) perspectives on SCP content and delivery.

**Conclusions:**

Our data point to the need to develop a process as well as written information for medically underserved breast cancer patients. An SCP document will not replace direct communication with providers about treatment, symptom management and transition, a communication that is missing in participating safety-net patients’ experiences of cancer care. Women turned to peer support and community-based organizations in the absence of information from providers.

**Implications for Cancer Survivors:**

“Clear and effective” communication of survivorship care for safety-net patients requires dedicated staff trained to address wide-ranging information needs and uncertainties.

## Background

The Institute of Medicine (IOM) and National Research Council 2005 report, *From Cancer Patient to Cancer Survivor*: *Lost in Transition*, recommends that patients with cancer who are completing treatment be “provided with a comprehensive care summary and follow-up plan *that is clearly and effectively explained*”[1]. The 2012 American College of Surgeons Commission on Cancer “Cancer Program Standards: Ensuring Patient Centered Care” requires the development and implementation of care transition plans for all cancer survivors as a standard of care [2]. According to this standard, the survivorship care plan (SCP) should contain a record of care received, disease characteristics, and follow-up plan.

Despite the IOM’s recommendation, few care organizations have instituted standard SCPs. In a study of 53 NCI- designated and comprehensive cancer centers, Salz and colleagues found that 23 of the 53 cancer centers (43%) used SCPs for their breast cancer survivors, colorectal cancer survivors, or both [3]. Of these 23 institutions, 17 (74%) reported using SCPs only for breast cancer survivors, 2 (9%) used SCPs only for colorectal cancer survivors, and 4 (17%) used SCPs for both groups of survivors. Survivors and primary care providers were reported to welcome SCPs in the context of comprehensive breast cancer care. However, a qualitative study that included breast cancer survivors, oncology specialists, and primary care providers found that while participants felt that written survivorship care plans could be helpful, they would be insufficient to ease the transition from oncology to primary care [4]. Assessment of SCPs in these studies did not address literacy level.

Much research has explored cultural differences in survivorship experience and meaning [5–10]. A growing literature is applying these understandings to the development and implementation of SCPs with the aim of infusing SCPs with the perspectives of patients [11–13]. To date, this research suggests that existing SCP templates fail to address the information needs of specific population groups or to be particularly useful to patients as they transition from oncology to primary care [11–14]. Some of this research suggests that health literacy may be an important mitigating factor. For example, health literacy has been associated with dissatisfaction and regret about breast cancer treatment among Latino women [15]; and inequality of access to cancer information, educational materials, and information about medication among African American breast cancer survivors [16]. Preliminary research conducted by colleagues at Olive View-UCLA Medical Center, with their low literacy, low English proficiency patient population, for example, revealed that 45% (n = 35) of patients did not remember being given a commonly used treatment summary developed by the American Society of Clinical Oncology (ASCO) (http://www.asco.org/institute-quality/breast-cancer-treatment-plan-and-summary-resources), and only 8.5% of those who had received the ASCO treatment summary had shown it to another provider [17]. We are aware of no research to date that explores survivorship information needs among safety-net breast cancer patients of multiple ethnicities and across multiple languages. Further, the link between these information needs and developing approaches to survivorship care plans is understudied.

This study emerged from a community-university collaboration established to address the lack of adequate and appropriately framed information for low income, low health literacy, and low English proficient breast cancer survivors transitioning from oncology to primary care. The San Francisco Women’s Cancer Network (SFWCN) is a group of organizations working toward the preservation of vital safety net programs, leading to the provision of a continuum of high quality, comprehensive, compassionate care for women with cancer. SFWCN members serve ethnically, linguistically and culturally diverse breast cancer survivors, the majority of whom have received care at San Francisco General Hospital (SFGH). SFWCN members identified the need for adequate information resources, and partnered with Mt. Zion Breast Cancer Center (BCC) and SFGH to identify a process to meet this need. SFGH was chosen as a partner because the majority of women served by SFWCN organizations received their care there, and Mt. Zion BCC because of their previously developed survivorship resources and expertise. The partnership came together with the understanding that survivorship transition challenges cut across all populations experiencing breast cancer and SFWCN members believed that low literacy SCP materials would benefit women across education levels.

The objective of our collaboration was to conduct inductive research to identify informational and structural challenges to treatment and survivorship for safety-net breast cancer patients that could inform the content and delivery of appropriate and useful SCPs. When we use the term safety net we refer to providers who offer services to patients regardless of ability to pay, and whose patient mix includes substantial numbers of uninsured, Medicaid, and other vulnerable patients [18]. We were particularly interested in identifying content and delivery preferences that would support the care transition from oncology to primary care and ongoing learning in survivorship for low literacy, multi-lingual patients. The study was designed to include the perspectives of both providers (e.g. oncologists, nurse practitioners, patient navigators, and social workers) and patients [19]. Herein we report findings from six focus groups conducted in English, Spanish, Cantonese, Russian, and Tagalog with women who were at least two years and no more than 15 years from their diagnosis.

## Methods and Study Design

Our collaboration convened a meeting with SFWCN member organizations serving predominantly low health literacy African American, Latina, Chinese, Russian, Filipina, and White medically underserved breast cancer patients in the San Francisco Bay Area. In the initial meeting, SFWCN representatives explained the study, inclusion criteria, and worked with member organizations to establish acceptable expectations for each organization. These included providing a focus group facilitator, recruiting women who fit the inclusion criteria, providing space to conduct the focus group, and providing feedback on preliminary findings. Inclusion criteria were breast cancer diagnosis, low health literacy and/or low English proficiency (LEP), at least two years and no more than fifteen years since diagnosis, and self identify as African American, Latina, Chinese, Russian, White/Euro-American, or Filipina. The range of two to fifteen years allowed for a diverse range of perspectives on information needs at different points in the survivorship experience, but all post active treatment (e.g., chemotherapy and/or radiation). Conversations with Mt. Zion BCC providers (oncologist, nurse practitioner) identified multiple time-points (i.e., 2–3 years out, 5 years out, and 10–15 years out from diagnosis) as important transitional survivorship periods when inquiries about follow-up care after treatment and management of long-term side effects (e.g., lymphedema, post-mastectomy pain syndrome, chemo brain) emerge in both oncology and primary care settings.

We chose focus groups as our primary methodology because of its capability to elucidate cultural nuance and the comparative ease of sampling population subgroups. As research on the cultural dimensions of focus group methodology [20] and our own previous research [21–23] suggest, the group process allows people to listen and to formulate their views if they are not ready to do so initially. SFWCN member organizations recruited a convenience sample of 5 African American, 7 Latina, 9 Chinese, 4 Russian, 6 White, and 7 Filipina women for participation in six focus group interviews. To facilitate recruitment, participants were offered a small stipend ($40) for their time, effort, and travel expenses. Focus groups were separated by language (e.g. English, Spanish, Cantonese, Russian, or Tagalog) and race/ethnicity (e.g. African American, Latina, Chinese, Russian, Euro-American, and Filipina). These stratifications were driven by the community-based organizations facilitating the focus groups. For example, one SFWCN member organization provided cancer support to African American women, therefore they recruited participants from their members; another provided support to Filipinas and recruited participants from their members, and so on. Although focus groups were stratified by language and race/ethnicity, mixed race/ethnicity women were also invited to participate if they were both a client of the respective SFWCN member organization facilitating the focus group and were able to participate in the language in which the focus group was conducted.

Focus groups were facilitated by bilingual, bicultural community organization staff who had undergone a half-day training with the university partners. Focus group sessions were recorded with the explicit consent of participants. Participants signed a written consent form translated into their primary language (e.g. English, Spanish, Cantonese, Russian, or Tagalog) prior to the beginning of each focus group and completed a demographic questionnaire in which they self reported their ethnicity, age, stage at diagnosis, etc. While we originally intended to administer a health literacy measure, SFWCN members felt that such a measure was unnecessary and that, due to the low income and low education levels of their clients, administering such a measure would introduce a barrier to participation in open discussion. All research activity was reviewed and approved by the University of California, San Francisco Committee on Human Research. Professional transcriptionists translated and transcribed focus groups into English for coding and analysis [24]. To ensure consistency in meaning, bilingual research team members conducted spot checks of translated transcripts (e.g. listened to portions of the original audio recording while reading the translated portion).

Focus groups were conducted in community centers and community organization offices. The purpose of the groups was to generate discussion amongst group members about the transition to primary care (Sample question: Could you share your understanding of what was supposed to happen with your healthcare after treatment ended? What were your expectations?), access to information (Sample question: Can you tell me about the kinds of information you received when you left the breast clinic?), knowledge of treatment history (Sample question: Can you tell me how you keep track of your treatment history? Who has helped?), and perspectives on SCPs (Sample question: What should be included in an SCP? When should it be provided?)

We followed standard qualitative analysis techniques in this study, including iterative data review, multiple coders, and “member checking”[25]. Three members of the research team reviewed all transcripts from group interviews with a particular focus on narrative related to informational needs and suggestions for SCP content. One team member (Napoles) coded each transcript for concepts and themes, starting with an open coding approach using inductive codes developed from transcript content and deductive codes linked to the research focus and original aims. After coding three transcripts the initial list of codes was shared with the broader study team, discussed, and refined. The resulting list formed the basis of the codebook that was applied to the remaining three transcripts. While coding was ongoing, two members of the research team developed “summary documents” which highlighted new ideas and concepts, recurrent ideas and concepts, and patterns or themes noted across transcripts. These summary documents were shared among the team and discussed in group meetings. The main themes identified in this collaborative analysis process, ongoing throughout the qualitative data collection phase, were shared and discussed with SFWCN members and their responses were incorporated into the analysis.

## Results

### Participant characteristics

Table 1 details demographic characteristics of the 38 focus group participants. The mean age of the participants was 61 ± 9. Forty-one percent of the participants (41%) had either less than a high school education or had completed high school or GED with the remainder equally distributed among other educational attainment categories. About a third of the participants (36%) had an annual household income of less than a $10,000.

**Table 1:** Participant Demographic Summary Statistics.

Characteristic	n (%)
**No. of participants**	**38**
**Age**	
Mean age ± SD	61 ± 9
Min-max age	39–77
**Race/Ethnicity**	
Asian	9 (24)
African American/Black	5 (13)
Latino/Hispanic	7 (18)
Filipina	7 (18)
White/Caucasian	9 (24)
Mixed-race	1 (3)
**Primary language**	
English	11 (29)
Chinese	9 (24)
Russian	4 (11)
Spanish	7 (18)
Tagalog	7 (18)
**Education**	
Less than high school	8 (22)
High school graduate or GED	7 (19)
Some college	7 (19)
2-year college degree (Associates)	2 (5)
4-year college degree (BA/BS)	8 (22)
Graduate degree	5 (14)
**Annual household income**	
$0 to $9,999	13 (36)
$10,000 to $14,999	6 (17)
$15,000 to $19,999	2 (6)
$20,000 to $34,999	8 (22)
$35,000 to $49,999	1 (3)
$50,000 to $74,999	2 (6)
$75,000 or more	3 (8)
Rather not say	1 (3)
**Employment**	
Full-time	6 (16)
Part-time	6 (16)
Not working	25 (68)
**Insurance**	
Private health insurance or HMO	7 (18)
MediCal	14 (37)
Medicare	7 (18)
Medicare & MediCal	7 (18)
Healthy San Francisco	3 (8)
**Age at breast cancer diagnosis**	
Mean age (SD)	56 ± 10
Min-max age	35–77
**Years since breast cancer diagnosis**	
Mean years (SD)	5± 3
Min-max years	2–13
**Breast cancer stage at diagnosis**	
Stage 0	2 (5)
Stage 1	12 (32)
Stage 2	12 (32)
Stage 3	10 (26)
Stage 4	1 (3)
I don’t know	1 (3)
**Surgery**	
Lumpectomy	18 (49)
Lumpectomy with reconstruction	3 (8)
Mastectomy	10 (27)
Mastectomy with reconstruction	6 (16)

### Qualitative analysis results

Analysis of the six focus group transcripts identified three themes: 1) the need for information and education on the transition between “active treatment” and “survivorship”; 2) information needed (and often not obtained) from providers, including information about screening, recurrence, side effects and pain, reconstruction, and healthy eating and physical activity; and 3) perspectives on SCP content and delivery. Together these themes point to the need for an SCP process as well as content that involves much more direct communication between survivors and providers. Structural issues such as the safety-net context of care (e.g. fleeting relationships with providers, long wait times, poor communication between oncology and primary care) were pervasive throughout the data. Therefore, we do not present these as a separate theme, but rather note that this context underlies and informs the information needs and experiences of care reported.

#### 1. Need for information and education on the transition between “active treatment” and “survivorship”

When asked to describe the transition from the end of breast cancer treatment to survivorship, women across the focus group interviews discussed how they often remained on ongoing treatments, like Tamoxifen or other hormonal therapies, despite ending active treatment. Therefore, while their providers considered them in survivorship, and no longer in active treatment, women were confused about the boundaries around these categories and assumed that treatment was treatment, whether ‘active’ or not (e.g. hormonal treatment). Women across all groups expressed confusion about definitions of survivorship. In the Euro-American group, one participant pondered the meaning of active treatment as possibly consonant with “initial treatment or intensive treatment.” Another participant personalized the confusion in her own experience of continued treatment side effects after being moved to ‘survivorship.’

I consider that my treatment is still ongoing because I’m still on the aromatase inhibitors. But I was very surprised to learn that my oncologist and the rest of the people at the Breast Center considered that I was now not in active treatment. I was particularly surprised because I had terrible, terrible problems with Tamoxifen and aromatase inhibitors…So I think it’s a huge mistake to say that “Oh, you’re not in active treatment anymore because you’re taking Tamoxifen or the aromatase inhibitors.” And I was very surprised to learn …that I was seeing my doctor less often, even though I had all these symptoms, especially because nobody explained to me what was going on…I did not realize that there was a change in protocol when you went over to the Tamoxifen and nobody explained that to me either. (Euro-American focus group)

Another member of this group summed up the discussion with, “I think we need new terms. Survivorship and active care may not be the most accurate of terms…Because survivorship implies that it’s over and it’s not over.” Those participants who did have an understanding of survivorship received this information from community organizations (e.g. SFWCN organizations serving African American, Spanish-, Cantonese- and Tagalog-speaking cancer patients) that hosted breast cancer support groups. Cantonese-speaking participants described support groups as almost taking the place of an SCP or follow-up from doctors, because they learned about a variety of issues they struggled with in these groups. African American participants described a range of information sources regarding survivorship. None had received written information on what to expect in survivorship or in the care transition from their oncologist or cancer care team. All reported relying on their support group for information. Several mentioned the value of the American Cancer Society binder they were given by their cancer care team. As one participant stated, “it [ACS binder] was wonderful. It had everything you needed to know about your treatment, the drugs you were given…But it was just about the treatment. Nothing about after.” When asked if anyone walked her through the information she responded,

No, they just handed it to me and told me it would be my bible. That it had everything I needed to know about each procedure, each drug, what might happen…But I never read it. I just took it and put it aside. Months later, when all this was happening with the neuropathy and everything I read through it and saw ‘ahhh’ so that’s why… But you see, I don’t read manuals. I like to get my information from someone. If they had talked me through it, I would have listened. But just to read, I’m not going to do that. (African American Focus Group)

Spanish-speaking participants mentioned “graduation” from the breast cancer clinic, but commented that it was not accompanied by any substantive information about survivorship or what to expect or do/not do as a survivor. A member of the Russian-speaking group mentioned that the transition into survivorship was “very scary to be left without the doctor.” When told to come back in six months after she finished chemo/radiation, this participant “felt fear, hopelessness, without understanding where to go, what to do.” Another stated, “nobody explained anything to me; to this day I’m clueless what to do and where to go.” A Chinese-speaking patient reported, “When I finished my cancer treatment, I felt very anxious. I had many questions. For example, do I keep taking medications and when will the cancer come back?” An African American participant reported fearing returning to her primary care doctor, who she blamed for not finding her cancer earlier. She had been seeing the same primary care provider for thirty-one years and had never undergone a clinical breast exam, nor was she “pushed” to get a mammogram despite being out of compliance.

In addition to a lack of communication between cancer care teams and patients, participants across focus groups characterized the communication between their cancer care team and PCP as suboptimal and in some cases, nonexistent. A Cantonese-speaking participant explained, “I feel like the whole [breast cancer] treatment process was disconnected from my primary care physician. My primary care physician was not involved at all.” Another Chinese participant agreed, “My primary care physician never asked me anything about my cancer. He didn’t ask me about the medications that I had been taking, the X-rays that I did, or the wound from my surgery. I just went back because I had the flu.” Other women who had stopped seeing a PCP during their cancer treatment found it challenging to navigate finding another PCP who could care for them as breast cancer survivors. As one Latina participant described, “I don't know [if I have a primary care provider] now. Currently, I am lost. I don't know what I am going to do because I have changed healthcare plans and I have been all over the place because I had a million surgeries.”

#### 2. Information needed (and often not received) from providers

Highlighting inadequate information sharing between and among providers, women also called for better communication between patients and providers. Five primary areas of information deficit emerged from our analysis across focus groups: screening, recurrence, side effects and pain, reconstruction, and healthy eating/physical activity.

Participants reported that they, and often their primary care doctors, were confused about the level of screening and monitoring they should expect in survivorship. A Filipina participant stated, “Okay, what exam to take, dates, wait, updates…I just found out now, after seven years, that you need to do a bone scan, liver scan, lung scan…” A Latina participant commented, “For example, the doctor, her primary care doctor, depending on how her blood is, they should do a blood check, do a cholesterol check, the bones, everything, calcium levels, and a general check-up of all of that.” These uncertainties were also expressed by a Chinese participant, “It’s been two years since I completed my treatment. From what the others have said, it seems like we should be going back for a breast exam once every year but it’s been two years and they haven’t contacted me yet.” The anxiety underlying these concerns about adequate monitoring amplified fears and concerns about recurrence.

Leaving the breast clinic and ending active treatment created new worries about recurrence and feeling “unprotected.” As women became long-term survivors and were farther away from their diagnosis, they became more worried that their cancer could come back. As one Chinese participant explained, “When I completed my cancer treatment, I felt very anxious… I was very worried that [the cancer] would relapse at any time.” Due to these anxieties, women called for more knowledge about what symptoms signify recurrence and “how to react”. As one Chinese participant explained, “Yes, [it will be helpful to have information] to warn us about conditions that we may come across that can be an early warning of a relapse of cancer so we can be more alert for these signals.” A Filipina participant stated, “Because after what happened, there were times that my right breast was in pain. I wonder why it would hurt and I start thinking I might have cancer again.”

Some participants felt their providers were not truthful about the length and severity of side effects, especially hormonal treatments. As one Russian participant explained, “[The doctor] assured me my side effects will go away in 18 months; it’s been four years. I still have side effects from chemo treatment.” A Euro-American participant agreed, “I didn't even bother to tell my oncologist because she had said there were no side effects so this couldn't be the Tamoxifen. It must be something else. And the GI doc said, ‘Oh, it’s because you’re taking Tamoxifen.’ Went to see the cardiologist, same thing. ‘Oh, it’s because you’re taking Tamoxifen.’” Other participants called for providers to proactively check in with patients about side effects rather than waiting for patients to complain. As another Euro-American participant described, “I found the doctors and nurses at the Breast Cancer Center really value stoicism. It became clear that not complaining and toughing it out were considered admirable qualities.”

Lymphedema was one of the most prevalent side effects experienced by participants. As one Chinese participant noted, “After my chemotherapy, my lymphedema is still giving me discomfort. I hope that new research can find ways to alleviate some of the sufferings that future breast cancer patients have to endure.” Another Chinese participant was unhappy with the lymphedema care she received saying, “[The doctor] just told me to do physical therapy for my lymphedema. I did that for half a year and didn’t feel any improvements. Every time I go there, [the physical therapist] would just measure me. I wanted him to apply some pressure around the area to loosen me up but he only measured me.”

Another area of information deficit that emerged from the focus groups was around breast reconstruction: what the alternatives are, when to have it, and how to care for oneself after surgery. As one Euro-American participant described,

…what I’d really like to see is some protocol about guiding you after you’ve had breast cancer after you know, you got drains and all this stuff and then you don’t have the drains. And then it’s kind of like ‘Oh, here’s your bra.’ And then it’s like nothing. There’s nothing about…if you want to deal with the scar or if you want to make sure your skin doesn't stretch or you know, stuff about reconstruction. It’s a black hole.

Women stressed the importance of making healthy lifestyle choices when living with cancer and expressed that they wanted their doctor or support group [for those who attended support groups] to “provide recommendations.” As one Chinese participant explained, “It would help if someone can teach us what to eat or what exercises to do to relieve the pain. My arms and legs have the same numbing sensation. It’s even painful to walk.” Latina participants requested information on yoga and other forms of low-impact exercise.

An African American participant shared her experience with severe neuropathy and subsequent attempts to exercise. She wanted to start exercising but was unable to feel things when she touched them. No one had given her direction on what was okay or not to do. She thought water aerobics would be a good option. If she touched the floor of the pool, she would not feel it with her feet, but thought that would be okay. She could move through the water. So she did that for a while. Then she thought ‘why not add weights?’ She started using the arm weights in the water, not realizing that this could cause a hernia. No one had told her that could happen.

Participants also discussed the importance of knowing what to eat “the moment that you are diagnosed with cancer.” Women in the Tagalog-speaking focus group stressed the importance of knowing what foods to avoid and likewise, what foods are “cancer-fighting.” Women in the Spanish- and Cantonese-speaking focus groups discussed the importance of nutritional supplements such as Vitamin D to help regulate calcium levels.

#### 3. Perspectives on SCP content and delivery

Due to the confusion of transitioning from cancer treatment to survivorship and a cancer care team to a primary care provider, women had many questions, some of which were not obvious at the time; they wished a SCP would better equip them with the questions to ask one’s doctor. In addition, women across focus groups suggested the SCP should include referrals to PCPs knowledgeable about breast cancer and side effects. As one Euro-American participant explained, “Like, ‘Okay, we’re transitioning you about not seeing your oncologist all the time. Here’s a list of people you can see that know stuff about breast cancer.’ Like that simple thing would be huge, you know.”

In addition to information on how to prevent breast cancer from recurring and “monitor conditions for any changes,” women requested information on how to manage side effects and minimize pain. Participants described experiencing several side effects of both active and hormonal treatment such as memory loss, loss of movement and coordination, hair loss, dry skin, joint pain, numbness, and fatigue, calling for a post-cancer treatment guide and acknowledgment of how their bodies, emotions, and everyday lives had changed. As one Chinese participant said, “I told my doctor that sometimes my fingers are so stiff that I couldn’t even hold my chopsticks.” One Russian participant described how the side effects and pain were unbearable, “I was so scared that I might die, I was ready to overdose with my medications—just not to feel pain; it was very scary.”

Several women in the Cantonese-speaking focus group discussed the importance of receiving information on hereditary breast cancer as part of the SCP. As one Chinese participant explained, “As mothers, we worry about our daughters. Some of these illnesses are hereditary. I’m fine with suffering the pain, but I worry that I might pass it on to the next generation.” Another participant agreed, “After my surgery, I asked my doctor if my cancer would be passed on to my children since I was not aware that I had a family history of cancer.” Similarly, women in both the Spanish- and Cantonese-speaking focus groups requested guidance on how to talk with family about living with cancer. One Latina participant explained, “…sometimes I would get angry and I would tell [my family], ‘The fact is that you do not know. It's that you are not going through what I am going through. I feel like this.’ You feel like they reject you or sometimes they make you feel worse than you feel because they don't give you support.” Another participant chimed in with agreement, “We all feel like her. We have suffered, we understand each other, but other people that haven't had it don't understand you.” A Chinese participant noted that her friend and breast cancer support group gave her strength and guidance whereas “At home, nobody can really advise me on how to cope with breast cancer.”

In addition to these topics, women requested that the SCP include information on self-care strategies related to diet, nutrition and exercise. Smoking cessation was also mentioned in the Tagalog-speaking focus group. Participants recommended that SCP include the tools to help one eat well, maintain a healthy weight, and get regular exercise.

Participants suggested that having a dedicated person in the clinic to discuss the transition between the first (e.g. active) and second (e.g. hormonal) stages of treatment would be extremely helpful. One participant in the Euro American group stated,

I hope that there can be a department that specifically focuses on breast cancer patients. When I completed my cancer treatment, I felt very anxious. I had many questions, for example, do I keep taking medications and when will the cancer come back? I was very worried that [the cancer] would relapse at any time …Sometimes when we bring these problems up to our primary care physician, they actually just advise us to speak to our oncologists. Therefore, it would be very helpful to have a place where we can go to get information and to get an understanding so that we don’t have to keep worrying about different things.

Women identified various time points when the kinds of information they would like to see in an SCP should be delivered. These included at transition points during their treatment experience (e.g. when side effects start to emerge), at the transition between “active treatment” and “survivorship” (e.g. at the point of “graduation” from the breast clinic for those seen at the public hospital), and at subsequent points when needed (e.g. the “department” mentioned above, where women can go with questions as problems arise in survivorship).

While we probed about the format(s) an SCP might take (e.g. single page treatment summary, information sheets, online resource, etc.) participants consistently shifted this conversation back to information needs and the benefits they had received from community based organizations and support groups. When written information was discussed, preference was expressed for both clear (e.g. lay language) English and the non-English language spoken by group participants.

## Discussion

In order to ensure the overall well being of cancer survivors, experts and policy makers agree that a comprehensive and coordinated approach to post-treatment care is required [26]. Recommended steps have been outlined by the IOM and LIVESTRONG in their brief: The Essential Elements of Survivorship Care [27], and the Commission on Cancer (COC) standard 3.3 requires a staggered implementation of the provision of SCPs between 2016 (to 25% of patients) and 2018 (to 75% of patients) [24]. However, challenges in addressing these elements of post-treatment care in a safety-net setting with non-English speaking and limited English proficiency (LEP) breast cancer patients has received little attention. A recent study of SCP preferences among Chinese American breast cancer survivors found that the women interviewed would prefer reviewing the treatment summary in person with a provider, and that follow up written information in lay language in English and Chinese would be acceptable [11]. Preferred content was similar to that identified in the data presented herein, with the addition of requests for information on Traditional Chinese Medicine. Burg’s findings from research with African American breast cancer survivors resonates with the findings reported in this study. Women in her research received variable amounts of information about their cancer treatments [14] and were discontent with the race-specific information they received. The American Society of Clinical Oncology’s (ASCO) breast cancer survivorship care plan in use at the time of the study was viewed as too technical and lacked detailed information on side effects and self-care [14]. Other research with minority breast cancer survivors reports that SCP templates are too generic [13,14,28]. In a focus group study where male and female cancer survivors reviewed an SCP template for colorectal cancer, Hewitt et al. reported that participants preferred SCPs that were more personalized, tailoring the treatment plan to the individual, and were written in layman’s terms [28]. In a recent study of the responsiveness of SCPs to the needs of African American breast cancer survivors, Ashing-Giwa and colleagues held consensus meetings with survivors and advocates to identify culturally responsive SCP content and domains [12]. Recommendations included documentation of all co-morbidities and medications regardless of relationship to cancer, referrals for cancer-related providers, and culturally informed health advisories. The authors concluded that the available SCP template lacked adequate content on health history, co-morbidity, health promotion, and functioning [12]. Further, they argue that the emerging science and implementation of SCPs are void of patient input. Overall, this research suggests gaps in the ability of existing SCP templates to address the information needs of specific population groups or to be particularly useful to patients as they transition from oncology to primary care [11–14]. The study reported here is the first that we are aware of that includes perspectives of ethnically and linguistically diverse safety-net breast cancer patients on SCP content and delivery. It demonstrates the value of and need for direct communication between patients and providers about survivorship care, thus highlighting the need for conceptualizing the SCP as a process with information provided at multiple time points and via various mechanisms (e.g. low literacy information sheets in language, direct communication, etc.).

SCPs have been recognized as having strong face validity [1]. In addition, cancer survivors have expressed enthusiastic support for SCPs [28,29]. However, the promise of an SCP as a quality improvement tool is dependent in large part on system level issues [13,14]. Oncologists often do not have the time to create individualized SCPs or to discuss the content with their patients. Despite research reporting the need to develop cancer survivorship educational strategies that are responsive to the needs of specific populations, such as those included in this study, and the psychosocial profiles that motivate requests for extensive follow up guidance [13], adoption of SCPs and development of processes for their delivery have been slow. This is likely due to the resources required for their development, lack of provider buy-in for their utility, and reimbursement issues [13]. Currently, there are no clear mechanisms for reimbursement for the time it would take oncologists or other members of the oncology team to provide elemental survivorship information such as that requested by our participants, or to spend time coordinating the flow of information between their own clinics and the PCP.

## Limitations

This is preliminary work, and more needs to be done. While we note that the majority of our participants were low health literacy and LEP, we did not administer a health literacy measure in the course of this study. This decision was made in partnership with the SFWCN member organizations. These community organizations serve largely low health literacy and LEP breast cancer survivors living in poverty or precarity. Because they recruited from their organization membership, they felt that a health literacy measure was unnecessary and would introduce another barrier to participation in open discussion.

In the course of the focus groups, our participants tended to shift the conversation away from SCP content and format and back to descriptions of information needs, uncontrolled side effects and symptoms, and being generally lost in the system. The idea of a transition from cancer care to PCP, for many, was not something previously seriously considered. Perhaps because many were dealing with multiple co-morbidities, poverty, and insecure housing, and their experiences of cancer care were fraught with miscommunications, lack of information, and other structural barriers, the perceptions shared about survivorship and the SCP reflected a desire for improved communication and support.

### Conclusion

Our data point to the need to develop a process as well as low literacy written information in multiple languages. An SCP document will not replace direct communication with providers about treatment, symptom management and transition, a communication that is missing in the experiences of cancer care reported in this study. As noted in our focus groups, women turned to peer support and community-based organizations in the absence of information from providers. Relatedly, our data suggest there is less interest in a treatment summary, and more interest in information and education around what to do and how to manage survivorship. This speaks to the question of defining survivorship that emerged strongly in our data, but has not been addressed in the burgeoning literature on SCP development and implementation. Our inductive methods raise new issues, and suggest the need for dedicated survivorship coordination and navigation, in addition to follow up written information, available in low literacy formats and in multiple languages. Further research and action on implementation of SCP will need to address the structural barriers to implementation as well as the form and content of the survivorship care planning process to address the needs of diverse survivors.
